# The prophylactic effect of *Acetobacter syzygii* probiotic species against squamous cell carcinoma

**DOI:** 10.15171/joddd.2017.037

**Published:** 2017-12-13

**Authors:** Zahra Aghazadeh, Firoz Pouralibaba, Ahmad Yari Khosroushahi

**Affiliations:** ^1^Dental and Periodontal Research Center, Tabriz University of Medical Sciences, Tabriz, Iran; ^2^Department of Oral Medicine, Faculty of Dentistry, Tabriz University of Medical Sciences, Tabriz, Iran; ^3^Drug Applied Research Center, Tabriz University of Medical Sciences, Tabriz, Iran; ^4^Department of Pharmacognosy, Faculty of Pharmacy, Tabriz University of Medical Sciences, Tabriz, Iran

**Keywords:** Apoptosis, anticancer, probiotics, squamous cell carcinoma

## Abstract

***Background.*** Squamous cell carcinoma is a prevalent carcinoma of the oral cavity. Recently anti-proliferative effect of probiotics has been considered and assessed against different cancers. The aim of this study was to evaluate the cytotoxicity of *Acetobacter syzygii* strain supernatant on KB human oral cancer cell line and KDR human epithelial normal cell line.

***Methods.*** The cytotoxicity assessments were performed through 3-(4,5-dimethylthiazol-2-yl)-2,5-diphenyltetrazolium bromide (MTT) as well as through qualitative (4',6-diamidino-2-phenylindole staining) and quantitative (flow cytometry assessments using the BD Biosciences Annexin V-FITC Kit) evaluations of apoptosis.

***Results.*** *A. syzygii* secretions exhibited significant cytotoxicity against KB cancer cell lines similar to cisplatin (75.7% apoptosis) while the rate of apoptosis in KDR normal cells was only 9.36%. The prophylactic effects of Lactobacillus acidophilus (PTCC 1643), as a reference bacterium, was similar to *A. syzygii*, indicating beneficial effects of useful bacteria on prevention of oral diseases.

***Conclusion.*** The anticancer bioactivity of *A. syzygii* strain secretions depends on the induction of apoptosis in KB cancer cells. However, several investigations should be conducted to precisely determine effective compounds to be used as anticancer therapeutics in the future.

## Introduction

Squamous cell carcinoma of the oral cavity comprises approximately 94% of all malignancies.^1^ The death rate of mouth and oropharynx cancers has been reported to be 9500 per year, whereas the survival rate has been reported to be 50% in 5 years.^2^ The incidence of oral cancer in Asian countries, particularly in East Asia, is significantly different from that in western countries. Oral cancer has ranked the first for men all over the world.^3^ Nowadays it is obvious that cancer of the oral cavity is a multifactorial disease like other cancers, which develops by various factors such as lifestyle, habits and behaviors. Recently, dietary factors, such as iron deficiency and infectious agents like human papillomavirus have been implicated as effective factors in the development of oral diseases.^4^

Although few studies have assessed microbial compositions in subjects with squamous cell carcinoma (SCC), recent studies have shown that the microbial flora of patients and healthy individuals are different.^5^ On the other hand, various factors such as diet, age and smoking can also affect the microbial composition and development of SCC.^6^ Although the carcinogenesis of oral flora has not been proved, cytokines and other secretory mediators of bacteria can change cellular metabolic pathways, leading to malignant transformation;^7,8^ thus, high incidence of oral cancer in patients with poor oral hygiene can be a proof for increasing oral tumors.^9^ Studies on other parts of the gastrointestinal tract, including colon, have shown that some intestinal flora play important roles in carcinogenesis by production of enzymes such as glycosidase, azoreductase, nitroreductase and beta galactosidase, which transform the pre-carcinogens to active carcinogens.^10,11^ However, preliminary data have shown that probiotic products may be protective against the carcinogenic activity of these compounds; accordingly human studies have demonstrated that consuming *Lactobacillus acidophilus* and *Lactobacillus casei* reduces the level of the enzymes above in volunteers.^11^

Various studies have evaluated the effect of probiotics on different aspects of oral health as well. Studies have shown that consuming probiotics reduces *Streptococcus mutans* counts significantly, preventing tooth decay.^12,13^ The results of studies on periodontal patients have shown that consumption of probiotics can improve periodontitis and help eliminate halitosis.^14,15^ Additionally, probiotics are effective for the treatment of patients with oral cndidiasis.^16^ Despite numerous studies on the effect of probiotics on oral health, the effect of probiotics on oral cancer (squamous cell carcinoma) has not been studied.

In this study, the effect of *Acidobacter syzygii*, isolated from the regional traditional dairy products,^17,18^ was studied on the oral cavity cancer cells (KB) and on normal epithelial cells (KDR). The aim of this study was to determine the bioactivity and biofunctionality of bacteria for their probable use as pharmaceutical agents.

## Materials and Methods

### Cell line growth

Human oral cancer (KB) and human normal epithelial (KDR) cell lines were gained from Pastor Institute, Tehran, Iran. All the cancerous/normal cells were cultured in RPMI-1640 medium supplemented with 100 IU/mL of penicillin, 10 µg/mL of streptomycin, and 10% (v/v) of fetal bovine serum. All the cells were incubated at 37°C, 95% humidity and 5% CO_2_.

### Bacteria-secreted metabolite preparation

*Acidobacter syzygii* and *Lactobacillus acidophilus* (PTCC 1643), as a reference bacterium, were cultured in De Man, Rogosa and Sharpe (MRS) medium (Merck, Darmstadt, Germany) for 24 h. In order to isolate active bacterial supernatants, the cell cultures were centrifuged at 10,000 ×g for 10 minutes at 4°C. Since the pH of cell-free supernatants was low, the pH was neutralized and adjusted at the physiological pH (pH 7.2). Then, the pH-adjusted bacterial supernatants were carefully sterilized by filtering through Nalgene syringe filter units (0.22 µm) before treating cancerous or normal cells.

### Pronase and lipase test to identify the nature of the supernatant

To assess the nature of the actively secreted metabolites, pronase and lipase (Roche Applied Science, Penzberg, Germany) were separately added to the supernatants at a concentration of approximately 20 U/mL; then the mixture was incubated at 37°C for 30 minutes for protein and lipid content digestion. After the deactivation of pronase and lipase by heat denaturation, the treated supernatants were evaluated by MTT assay, similar to the untreated supernatants.

### MTT assay

The cancer and normal cell lines were seeded on a 96-well microplate (Nunc, Roskilde, Denmark) with a cell density of 1.2×10^4^ cells/each well. After 24 hours of seeding (40–60% confluency), 50 µL of filtered supernatants in different concentrations (10‒100 µg/mL) and LC_50_ concentration of cisplatin (positive control) were added for each well to achieve a total volume of 200 µL. All the treated/untreated cells were then incubated for 24 hours similar to the growth condition. After replacing the previous medium with fresh medium, 50 µL of the MTT solution (5 mg/mL in PBS) was added to each microplate well. Then the plates were incubated for 4 hours in 5% CO_2_ at 37°C under dark conditions. After removing MTT-containing media, crystals of Formazan created with MTT-exposed live cells were deleted by adding 200 µL of dimethyl sulphoxide (DMSO) and 25 µL of Sorenson’s glycine buffer (0.1 M glycine and 0.1 M NaCl, pH=10.5) into each well. Then the wells were incubated at 37°C while being gently shaken for 20 minutes. The adsorption of each well was measured with a Quant ELISA Reader (Biotek, ELx 800, Winooski, VT, USA) at 570 nm.

### Apoptosis assay

#### 2.5.1. 4',6-diamidino-2-phenylindole (DAPI) staining

To detect apoptotic cells, the cells (1.2×10^4^ cells/mL) were administered into each well of a 6-well culture plate and incubated under growth conditions. After reaching 50‒60% confluence, the cells were treated with LC_50_ concentration of sterile supernatant and cisplatin for 24 hours. The treated cells were washed away with pre-warmed tissue culture media and fixed by freshly prepared fixative solution (pre-warmed RPMI, containing 4% formaldehyde) and were permeabilized with PBS, containing 0.1% Triton X-100 for 5 minutes at 37°C. Afterwards, the permeabilized cells were stained with 50 µL of Dapi dye (1:2000 Dilution) per well for 3 minutes under incubation at room temperature. The processed cells were washed with PBS (pH=7.2) three times and utilized for apoptosis assessments by fluorescent microscopy (BX63, Olympus, Japan) equipped with U-MWU2 fluorescence filter (excitation filter BP 330-385, dichromatic mirror DM 400, emission filter LP 420).

### Flow cytometry

Flow cytometry assessments were performed on the KB and KDR treated/untreated control cells for a period of 24 hours using the BD Biosciences Annexin V-FITC Kit (San Jose, CA, USA). The detached cells by trypsin were separated from their supernatant by centrifugation at 2000 ×g for 5 minutes at 28°C. The cell pellets were re-suspended in one time-binding buffer at a concentration of 1×10^6^ cells/mL and transferred to new tubes (5 mL). Then, 100 µL of the cell suspension was mixed with 10 µL of propidium iodide (PI) solution and 5 µL of Annexin V-FITC. Thereafter, the cells were placed in 5 mL of culture tubes for 15 minutes at room temperature under dark conditions. A total of 400 µL of the binding buffer (one time) was added again to each tube. The assessments were conducted with a FACS Calibur flow cytometer (BD Biosciences, San Jose, CA, USA). The analysis of 70,000 cells was accomplished at a rate of 1000 cells/s. Quadrant setting was conducted with the untreated cell line as the negative control. Data analysis was performed with Cell Quest Pro software program (BD Biosciences, San Jose, CA, USA).

### Statistical analysis

All the experiments were based on a completely random design, and data were analyzed with one-way ANOVA with an appropriate multiple-comparison test using SPSS 19.0 (SPSS Inc, IBM Company, Chicago, Illinois). All the graphs were prepared in Microsoft Office Excel. A P-value ≤0.01 (*P≤0.05) was considered statistically significant. Data were presented as the mean ± standard deviation of three measurements.

## Results

### Anti-proliferative effect on cancer and normal cell lines

**Figure 1:**
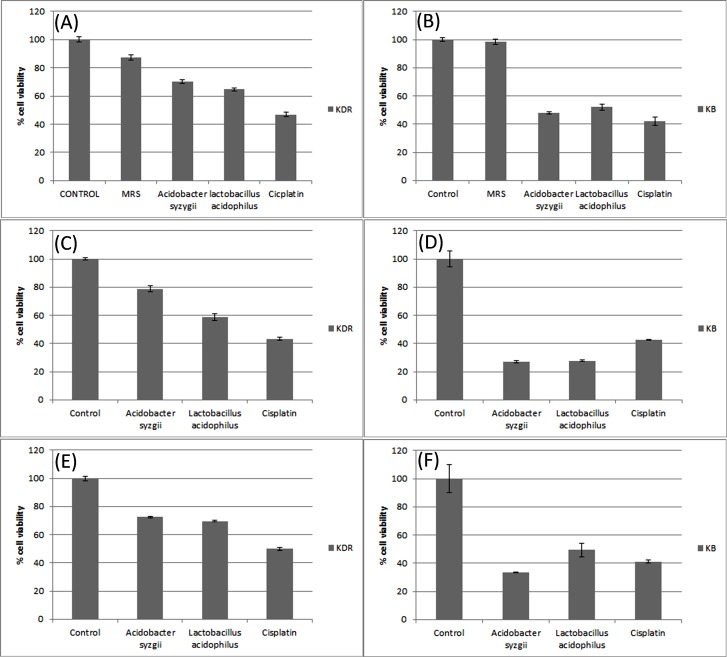
Effect of *Acidobacter syzigii* secretions on the proliferation of cancerous KB and KDR normal cell lines. *A. syzigii* secretions' final concentration: 60 µg/mL, cisplatin concentration: LC_50_ for each cell line. Incubation time was 24 hours. Data expressed as means and error bars represent standard deviation of means. Control: Untreated media were used as control. *Lactobacillus acidophilus* (PTCC) was used as the reference strain for comparison. Panels A and B: intact metabolite; panels C and D: pronase-treated metabolite; panels E and F: lipase-treated metabolite.

Screening of several concentrations of secretion metabolites (10‒100 µg/mL) revealed that anti-proliferative effect of bacteria on cancer cells is dose-dependent. Therefore, the LC_50_ concentration of each secretion metabolites by bacteria were firstly determined for KB cancer cells (*Acidobacter zysygii*: 60 µg/mL and Lactobacillus acidophilus: 10 µg/mL) and KDR normal cells (Acidobacter syzygii: 10 µg/mL and Lactobacillus acidophilus: 80 µg/mL) and all subsequent examinations were performed based on determined LC_50_. MTT assay findings showed a significant cell toxicity by intact secretion metabolites of Acidobacter syzygii on cancerous KB cell line similar to cisplatin, whereas the cytotoxic effect of these metabolites on KDR normal cells were even lower than cisplatin. The anti-proliferative effect of Acidobacter syzygii was higher than Lactobacillus acidophilus on KB but was lower on KDR cell lines (Figure 1, panels A and B). Based on our findings, the treatment of intact secretion metabolites by pronase and lipase cased an increasing cytotoxicity on KB cells indicating the protection effect of secreted proteins or lipids on KB cancer cells (Fig. 1, panels D and F). The pronase/lipase treatments did not show a significant effect on KDR cells compared to Kb cells (Figure 1, panels C and E).

### DAPI staining assessment

**Figure 2:**
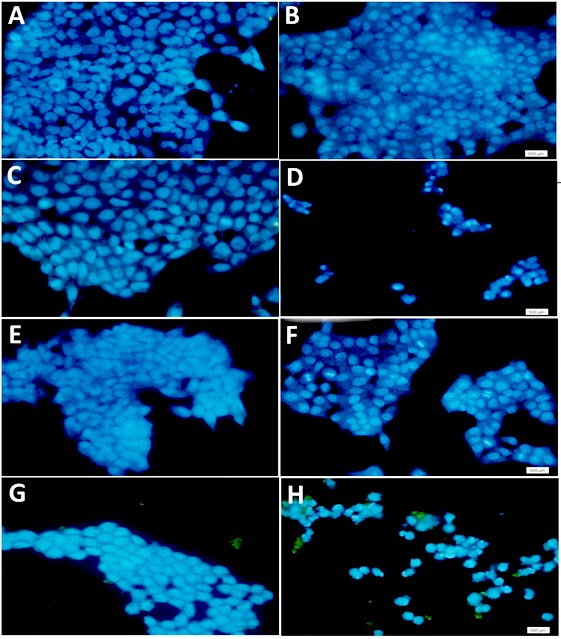
DAPI staining of treated (LC_50_)/untreated KB and KDR cancer cells for 24 hours. Panels A and B represent untreated KDR and KB cells, respectively; panels C and D represent *Acidobacter syzygii* supernatant-treated KDR and KB cells, respectively; panels E and F represent *Lactobacillus acidophilus*-treated KDR and KB cells, respectively; panels G and H represent cisplatin-treated KDR and KB cells, respectively.

The KB and KDR cell lines treated with secreted metabolites by *Acidobacter syzygii* (LC_50_) for 24 hours showed apoptosis symptoms, such as nucleus condensation or fragmentation; necrotic bodies were rarely observed. Hence, apoptosis was induced more often than necrosis. In all the treated groups, the number of apoptotic cells with fragmented and condensed nuclei were significantly higher than that of normal cells (showing intact nucleus). None of the distinctive apoptotic features were observed in the untreated KB/KDR cell lines (Figure 2, panels A and B). In the KDR cell line, all the treatments exhibited very low apoptosis symptoms (Figure 2, panels C, E, and G); rather, cell shrinkage (early apoptosis) and apoptotic bodies (late apoptosis) were the predominant apoptosis signals in the KB cells treated with *Acidobacter syzygii* secretions (LC_50_) after 24 hours (Figure 2 panels D, F, and H, ).

### Quantitative apoptosis assessment

**Figure 3:**
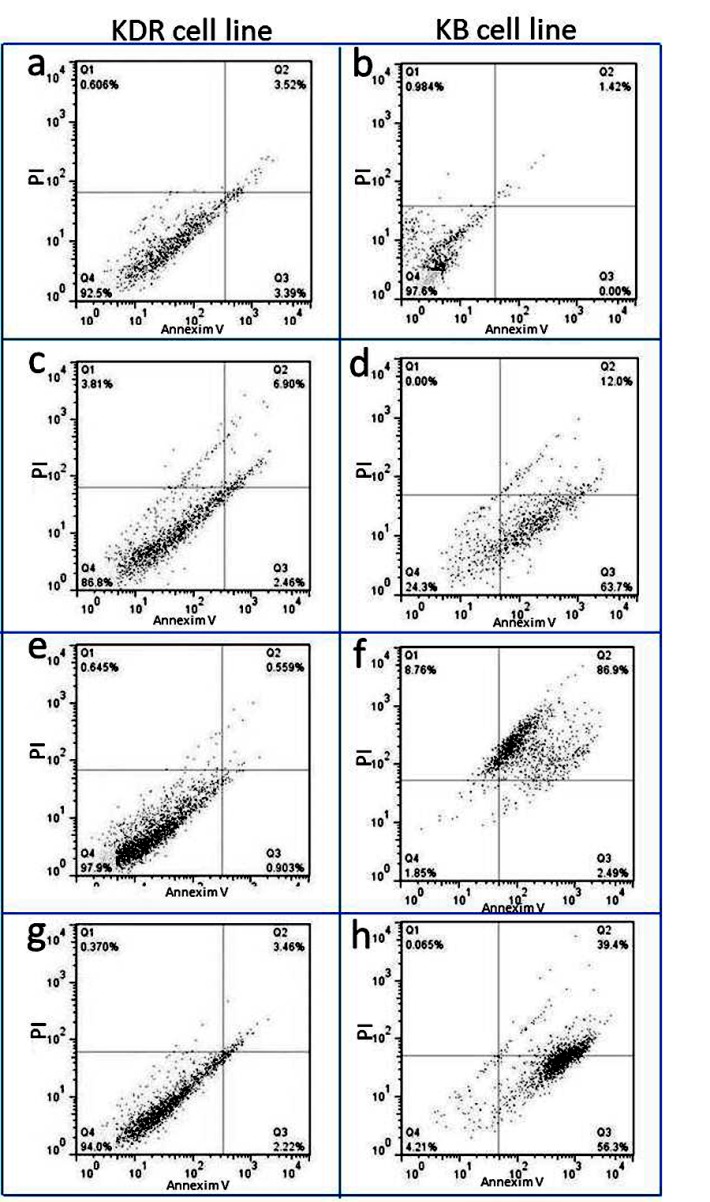
Flow cytometry analysis of treated (LC_50_)/untreated KDR and KB cells for 24 h. Panels a and b represent untreated cells; panels c and d represent *Acidobacter syzygii* supernatant-treated cells; panels e and f represent *Lactobacillus acidophilus*-treated cells; panels g and h represent cisplatin-treated cells.

*Acidobacter syzygii* secretions (LC_50_) on KB cell line induced 63.7% late apoptosis, 12% early apoptosis, and 0.0% necrosis for 24 hours (Figure 3, panel d). However, the untreated cells showed <1% cell necrosis and a total of 1.42% early apoptosis (Figure 3, panel b). *Lactobacillus acidophilus* secretions (LC_50_) on KB cell line, after 24 hours of treatment, exhibited 2.4% late apoptosis, 86.9% early apoptosis and 8.7% necrosis (Figure 3, panel f). Cisplatin (LC_50_) induced 56.3% late apoptosis, 39.4% early apoptosis and 0.06% necrosis on KB cell line for 24 hours (Figure 3, panel f). The results regarding KDR cell line were very different from KB. *Acidobacter syzygii* secretions (LC_50_) effects on KDR cell line consisted of 6.90% early apoptosis, 2.44% late apoptosis, and 3.81% necrosis for 24 hours (Figure 3, panel c) while the untreated cells exhibited a total of 3.52% early apoptosis, 3.39% exhibited late apoptosis and 0.60% necrosis for 24 hours (Figure 3 ,panel a).

*Lactobacillus acidophilus* secretions (LC_50_) induced 3.52% early apoptosis, 3.39% late apoptosis and 0.6% necrosis on KDR cell line for 24 hours (Figure 3, panel e). Apoptosis induction by cisplatin was approximately similar to the effects of *Acidobacter syzygii* secretions (LC_50_) on KDR cell line (Figure 3 ,panel g).

## Discussion

Probiotics are commonly used in many areas as pharmaceuticals and food supplements and in research. In recent years, the effects of probiotics in the biology, oncology and immunology fields have dramatically increased.^19,20^ Probiotics have recently been proposed at clinical, molecular and cellular levels in therapies for cancers, which usually involve surgery, radiotherapy or chemotherapy.^21^ For example, the results of research on *Bacillus polyfermenticus* showed that the bacterium is able to inhibit the growth of colon cancer cells, including HT-29, DLD-1 and Caco-2 cell lines.^22^

Molecular and experimental studies have shown anti-proliferative effects of probiotic bacteria. Studies on the efficacy of probiotics on different human cancers such as cervical, gastric, colon and breast cancer have shown positive results.^17^

In this study, the effect of metabolites secreted by *Acidobacter syzygii* were examined by cellular and molecular methods. The influence of altered concentrations of bacterial metabolites on the oral cancer KB line was assessed using MTT assay to study the anti-tumor effects. LC_50_ (60 μg/mL) was selected as the optimum concentration of *Acidobacter syzygii* while it was 10 μg/mL for *Lactobacillus acidophilus*.

The secretary metabolites were treated separately by lipase and protease enzymes in order to determine the type of effective substances. The treated metabolites were evaluated on the KB line again by MTT assay. Based on the results of this test, although the cancer cells were treated by lipase and protease, the anti-cancer effects of products which were produced by *Acidobacter syzygii* were not affected and therefore the effective metabolite has no lipid or protein structure and likely is in an exopolysaccharide structure type. Based on our findings, the lipid and proteins secreted by *Acidobacter syzygii* possess a protective effect on KB cancer cells since the pronase/lipase treatments heightened the cytotoxic effects of secreted metabolites on KB cells. The qualitative assessment of the effect of metabolites on cell lines, including normal and cancer cell lines, by DAPI staining demonstrated shrinkage and nuclear condensation in KB cancer cell lines, while there was no notable difference in apoptosis between the normal (KDR) and control cell lines (Figure 2). Clearly, the metabolites induce the apoptotic pathways more specifically in cancer cells rather than normal cells. The inhibition of cell growth by the metabolites of *Acidobacter syzygii*, DAPI staining results, was assessed using flow cytometry as a quantitative method.

The flow cytometry dot plot results, conducted in the treated and control groups, including healthy (KDR) and cancer (KB) cells lines, showed that bacterial secretary metabolites of *Acidobacter syzygii* led to the development of apoptosis in 75.7% of cancer cells, whereas apoptosis was only induced in 9.36% of normal cells. These results suggest that apoptosis is developed in oral cancer cells more than that in normal cells by bacterial secretary products.

*Lactobacillus acidophilus* induced apoptosis in the cancer cells KB (89.39%) and in normal cells (6.91%) compared to *Acidobacter syzygii*. Interestingly the induction of late apoptosis by *Acidobacter syzygii* was more than that by *Lactobacillus acidophilus* (63.7% for *A. syzygii* and 2.4% for *L. acidophilus*). Therefore the metabolites of *Acidobacter syzygii* induced apoptosis more quickly than *Lactobacillus acidophilus* in 24 hours in KB cancer cells. Additionally, the incidence of 8.7% necrosis by *Lactobacillus acidophilus* demonstrated the higher cytotoxicity compared to *Acidobacter syzygii* which caused 0% necrosis.

Cisplatin, which is applied as one of the most standard drugs for the treatment of patients with oral cancer, was considered as the positive control. The results of flow cytometry in groups treated with this drug demonstrated that apoptosis was induced at 95.7% for KB cancer cells and 6% for KDR normal cells. These results confirmed the results of *Lactobacillus acidophilus* (89.39%) tests and also revealed more apoptosis in cancer cells. Considering these findings, probiotic bacteria have acceptable ability to fight oral cancer cells. The results of studies on the effect of *Acidobacter syzygii* on other types of cancer cell lines (HeLa, MCF-7, AGS, and HT-29) revealed 14.84% necrosis and 33.01% apoptosis while based on the results of this study, this bacterium induced apoptosis in the oral cancer cell lines (KB) more effectively than the cancer cell lines of MCF-7 and Hela (56.38%)^18^ and it can be concluded that anti-cancer effects of probiotics are line-specific.

## Conclusion

*A. syzygii* isolated from curd, exhibiting typical probiotic properties, was utilized in this study to investigate the prophylactic effects on KB human oral cancer cell line. The cytotoxic finding showed that *A. syzygii* secretions exhibited acceptable anticancer activity on KB cancerous cell lines. In addition, based on the results of our study, *A. syzygii* secretion in comparison with cisplatin had similar anti-cancer activity. Moreover, *A. syzygii* secretions did not induce noticeable cytotoxic effect on KDR epithelial normal cell line. Finally, the physicochemical, structural and functional properties of effective anticancer substances (probably exopolysaccharides) for use in the treatment of cancer should be examined thoroughly.

## Acknowledgement

Authors would like to thank Drug Applied Research Center for supporting this project. This is a report of database from of PhD thesis entitled “Effect of probiotic bacteria on KB cell line” registered in Oral and Periodontal Research Center.

## Funding

This project was funded by Oral and Periodontal Research Center of Tabriz University of Medical Sciences.

## Competing interest

The authors declare no competing interests with regards to the authorship and/or publication of this article.

## Ethics approval

The study protocol was approved by the Ethics Committee of Tabriz University of Medical Sciences (TUMS) and was in compliance with Helsinki declaration.
